# Longitudinal course of hyperintensity on diffusion weighted imaging in adult-onset neuronal intranuclear inclusion disease patients

**DOI:** 10.3389/fneur.2023.1178307

**Published:** 2023-06-19

**Authors:** Dan Liu, Kai Chen, Song Tan, Long-Lin Yin, Mou Li, Yi-Shuang Wang

**Affiliations:** ^1^Department of Radiology, Sichuan Provincial People’s Hospital, University of Electronic Science and Technology of China, Chengdu, China; ^2^Department of Neurology, Sichuan Provincial People’s Hospital, University of Electronic Science and Technology of China, Chengdu, China

**Keywords:** neuronal intranuclear inclusion disease, diffusion weighted imaging, longitudinal changes, leukoencephalopathy, *NOTCH2NLC*

## Abstract

**Background:**

High signals on diffusion weighted imaging along the corticomedullary junction (CMJ) have demonstrated excellent diagnostic values for adult-onset neuronal intranuclear inclusion disease (NIID). However, the longitudinal course of diffusion weighted imaging high intensities in adult-onset NIID patients has rarely been investigated.

**Methods:**

We described four NIID cases that had been discovered using skin biopsy and *NOTCH2NLC* gene testing, after diffusion weighted imaging exhibiting the distinctive corticomedullary junction high signals. Then using complete MRI data from NIID patients, we analyzed the chronological diffusion weighted imaging alterations of those individuals that had been published in Pub Med.

**Results:**

We discussed 135 NIID cases with comprehensive MRI data, including our four cases, of whom 39 had follow-up outcomes. The following are the four primary diffusion weighted imaging dynamic change patterns: (1) high signal intensities in the corticomedullary junction were negative on diffusion weighted imaging even after an 11-year follow-up (7/39); (2) diffusion weighted imagings were initially negative but subsequently revealed typical findings (9/39); (3) high signal intensities vanished during follow-up (3/39); (4) diffusion weighted imagings were positive at first and developed in a step-by-step manner (20/39). We discovered that NIID lesions eventually damaged the deep white matter, which comprises the cerebral peduncles, brain stem, middle cerebellar peduncles, paravermal regions, and cerebellar white matter.

**Conclusion:**

The longitudinal dynamic changes in NIID of diffusion weighted imaging are highly complex. We find that there are four main patterns of dynamic changes on diffusion weighted imaging. Furthermore, as the disease progressed, NIID lesions eventually involved the deep white matter.

## Introduction

Neuronal intranuclear inclusion disease (NIID) is a rare progressive neurodegenerative disorder that is characterized by eosinophilic hyaline intranuclear inclusions in neuronal and specific somatic cells (1). In 1968, NIID was initially described (2). Based on the age of onset, NIID can be divided into three categories: infantile, juvenile, and adult (3). In addition, familial and sporadic cases have also been reported (4). NIID is a highly heterogeneous disease with a variety of symptoms, including cognitive decline, parkinsonism, cerebellar ataxia, peripheral neuropathy, and autonomic dysfunction (5). The presence of ubiquitin and ubiquitin-related proteins in the intranuclear inclusions in NIID indicates that the nuclear ubiquitin-proteasome system may play an important role (6). GGC repeat expansion (>66 times) in the 5′-untranslated region (5′UTR) of *NOTCH2NLC* was identified as the most common causative factor of NIID (7).

In 100 and 81% of sporadic and familial cases, respectively (8), a high-signal linear lesion in CMJ on DWI is a strong indicator for the diagnosis of adult-onset NIID, which frequently prompts doctors to perform a skin biopsy and a gene test for an antemortem diagnosis (9, 10). However, it remains unclear whether DWI characteristics appear before or after the clinical presentation of NIID. Few findings have been published on the long-term progression of DWI hyperintensities.

The purpose of this study was to analyze the longitudinal progression of DWI alterations in NIID patients and to investigate the longitudinal course of the lesion’s severity and location.

## Methods

### Subjects

First, we presented four NIID patients that had been identified using skin biopsies and *NOTCH2NLC* gene testings based on DWI characteristics. Then, using PubMed as our primary source, we conducted a literature review with an emphasis on the long-term outcomes of DWI in NIID. We searched for articles with phrases “neuronal intranuclear hyaline inclusion disease,” “intranuclear inclusion body disease,” or “neuronal intranuclear inclusion disease.” In addition to obtaining case studies and excerpts from certain original articles, references were systematically searched. We discovered 126 cases in English and 9 cases in Japanese. All images were reviewed by an experienced radiologist (with 10 years of specialized experience) who was blinded to the clinical information. Ethical approval was obtained from the Ethics Committee of Sichuan Provincial People’s Hospital and written informed consent was obtained from all the patients.

### Neuroimaging examination

T1WI with or without gadolinium enhancement, T2WI, FLAIR, and DWI were systematically reviewed. On DWI, curvilinear high-signal lesions in the CMJ were examined. Without segmentation of the brain lobes was performed.

## Results

### Clinical characteristics of our four NIID patients

#### Patient 1

An 18-year-old Chinese female experienced recurrent temporary numbness in the right lower limb, which gradually spread to the right upper limb and face over 3 years. The attacks, which lasted a few days, began once a year and gradually escalated in frequency to numerous strikes each year. The Mini-Mental State Examination score of 27/30 and the Montreal Cognitive Assessment score of 21/30 revealed severe cognitive impairment. The cerebrospinal fluid (CSF) examination was carried out and the results were unknown. Serum lactate levels were elevated (3.50 mmol/L) at rest and significantly increased (8.30 mmol/L) following the simplified serum lactate exercise test. Similar symptoms were also experienced by her mother (who was the second child in the family and died at the age of 40), elder sister, cousins, and three aunts. Coenzyme Q10 and riboflavin were used, but the symptoms did not significantly improve. Initially, based on cortical changes on brain MRI and a positive lactic acid test, mitochondrial encephalomyopathy, lactic acidosis, and stroke-like episodes (MELAS) was suspected. However, the DNA test failed to find any causative variants. Eosinophilic intranuclear inclusions in fibroblasts and ubiquitin-positive granules in fibroblasts and sweat gland cells were both observed in a skin sample. There was no enlarged CGG permutations found in the peripheral blood test for the fragile X chromosomal mental retardation gene (*FMR1*) in 5′UTR (30 times). The *NOTCH2NLC* gene in 5′UTR had 70 GGC repeats. She was subsequently identified as NIID.

#### Patient 2

A 28-year-old Chinese woman, the older sister of patient 1, experienced left and right cross headaches as well as recurrent migraines with no apparent cause. She experienced a dull, throbbing pain and blurry vision in her right eye. She then started vomiting and became speechless. Severe cognitive impairment was indicated by patient’s scores of the Mini-Mental State Examination (27/30) and the Montreal Cognitive Assessment (21/30). She was also first suspected of having MELAS. Family history was similar to the patient 1. The other data did not show any abnormalities. MELAS-related gene tests were unable to find any causative variants. The skin biopsy was then done, and the results showed eosinophilic intranuclear inclusions in fibroblasts and ubiquitin-positive granules in fibroblasts and sweat gland cells. The *FMR1* premutations CGG repeats were within the normal range (30 times). With 66 repeats, the *NOTCH2NLC* gene had an abnormal increase GGC repeats in the 5′UTR. In addition, patient 1 and 2’s father, their third aunt, and their fourth aunt all underwent serum *NOTCH2NLC* genetic testings with results of 26,19,60 repeats, respectively, and then their fourth aunt underwent *FMR1* genetic testing. The *FMR1* gene test did not show any enlarged CGG premutations (35 times). Their mother passed away at the age of 40 without undergoing genetic testing, their oldest aunt, and cousins all refused to do the related gene tests. As a result, the family cannot be adequately appraised of genetic analysis based on the information already available.

#### Patient 3

A 71-year-old Chinese man was unable to stand and hospitalized for 35 h with limb weakness without obvious causes, notably on the left side. He had a history of hypertension for more than 2 years and essential tremors in both hands for 7 years. He was initially diagnosed with cerebral hemorrhage. The results of Mini-Mental State Examination (23/30) and the Montreal Cognitive Assessment (16/30) both indicated severe cognitive impairment. No other members of the family have similar clinical symptoms. The cerebrospinal fluid (CSF) examination was carried out and the results were unknown. Then, after taking a skin sample, we found eosinophilic intranuclear inclusions in adipocytes, sweat gland cells, fibroblasts that were ubiquitin-positive. The *FMR1* gene revealed no expanded CGG premutations. The number of GGC repeats in *NOTCH2NLC* gene was 80.

#### Patient 4

A 64-year-old Chinese man with a history of diabetes was referred to our hospital because of the right hand tremors. Four years ago, the patient’s right hand began to tremble, and it worsened when he grew upset. A year ago, the patient also developed constipation, tremors in the left upper limb, and progressive weakening in both lower limbs. No one in his family has ever suffered a neurological condition. A diagnosis of Parkinson’s disease was made, however, levodopa and trihexyphenidyl did not alleviate the tremor. So drug use was voluntarily terminated. The patient was admitted to our hospital’s outpatient unit for further treatment. Skin biopsy showed ubiquitin-positive eosinophilic intranuclear inclusions in fibroblasts. The *FMR1* gene revealed no expanded CGG premutations. The number of GGC repeats in *NOTCH2NLC* gene was 118.

### Brain MRI of longitudinal findings of our patients

Table 1 provides a summary of the MRI findings for the four patients with NIID. Each patient had a minimum of two MRI scans of the brain.

**Table 1:** Summary of DWI findings of the NIID patients.

Case1		Case 2		Case 3		Case 4	
Time	DWI	Time	DWI	Time	DWI	Time	DWI
Onset	−	Onset	BCH+ CC+	Onset	NE	Onset	NE
7D	−	2Y	BCH++ CC+	1Y	NE	1.5Y	BCH+; CC+ IC+; CP+ MCP+; BS+ CH+; PV+
1Y	−	3Y	BCH+++; CC+ DWH+; EC+	2Y	NE	1.8Y	BCH+; CC+ IC+; CP+ MCP+; BS+ CH+; PV+
2Y	BF+ RP+ CC+	4Y	BCH+++; CC+ DWH+; EC+	2.5Y	NE		
2.5Y	BF++ RP+ CC+	4.3Y	BCH+++; CC+ DWH+; EC+	5.5Y	NE		
		4.5Y	BCH+++; CC+ DWH+; EC+	6Y	BCH+; EC+ DWH+; CH+; PV+		
		4.6Y	BCH+++; CC+ DWH+; EC+	8Y	BCH+; EC+ DWH+; CH+; PV+		

Time, Interval between two MRIs; BCH, bilateral cerebral hemispheres; BF, bilateral frontal lobe; RP, right parietal lobe; CC, corpus callosum; EC, external capsule; IC, internal capsule; CP, cerebral peduncle; BS, brain stem; PV, paravermis; MCP, middle cerebellar peduncle; CH, cerebellar hemisphere; NE, not examined.

#### Patient 1

**Figure 1:**
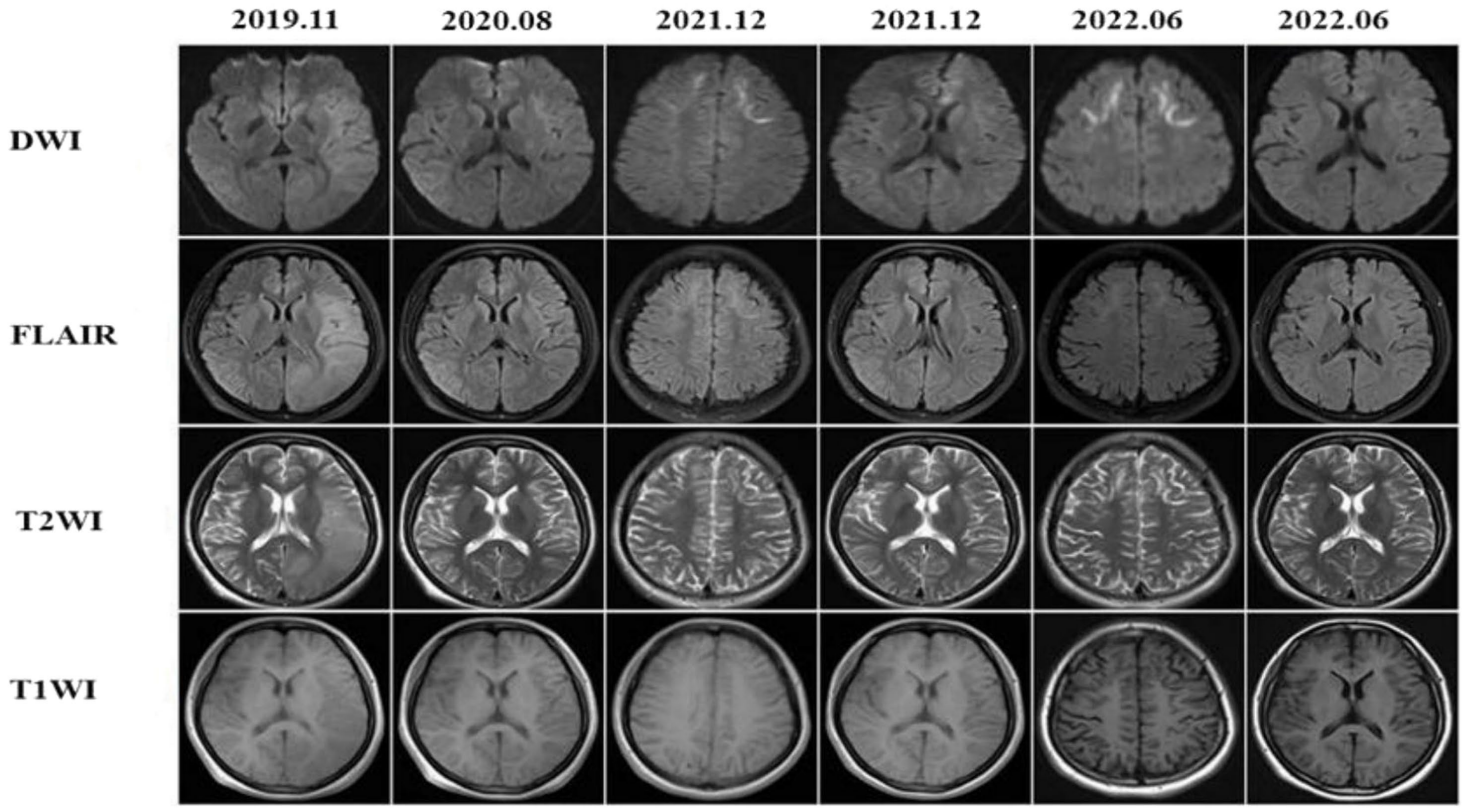
The patient presented with recurrent episodes of encephalitis with reversible cortical swelling on the left cerebral hemisphere in 2019 and the edema was completely recovered in 2020, but the typical high-intensities on DWI were observed at the bilateral frontal lobes, right parietal lobe and the corpus callosum in 2021. The lesions at the bilateral frontal lobes were more prominent in 2022.

After the first admission in 2019, the patient 1’s head MRI revealed left cerebral edema simultaneously with the emergence of encephalitic symptoms (Figure 1, 2019–11). The cerebral edema was not symmetrical on T2WI and FLAIR. A subsequent brain MRI in 2020 revealed that the cerebral edema had vanished completely. It is worth noting that neither high signals on DWI nor abnormal gadolinium enhancement were detected in 2019 or 2020. However, 2 years later, DWI indicated typical lesions in the bilateral frontal lobes, right parietal lobe, and corpus callosum. The right parietal lobe and corpus callosum lesions had not changed substantially after 6 months, whereas, the bilateral frontal lesions had progressed on DWI.

#### Patient 2

**Figure 2:**
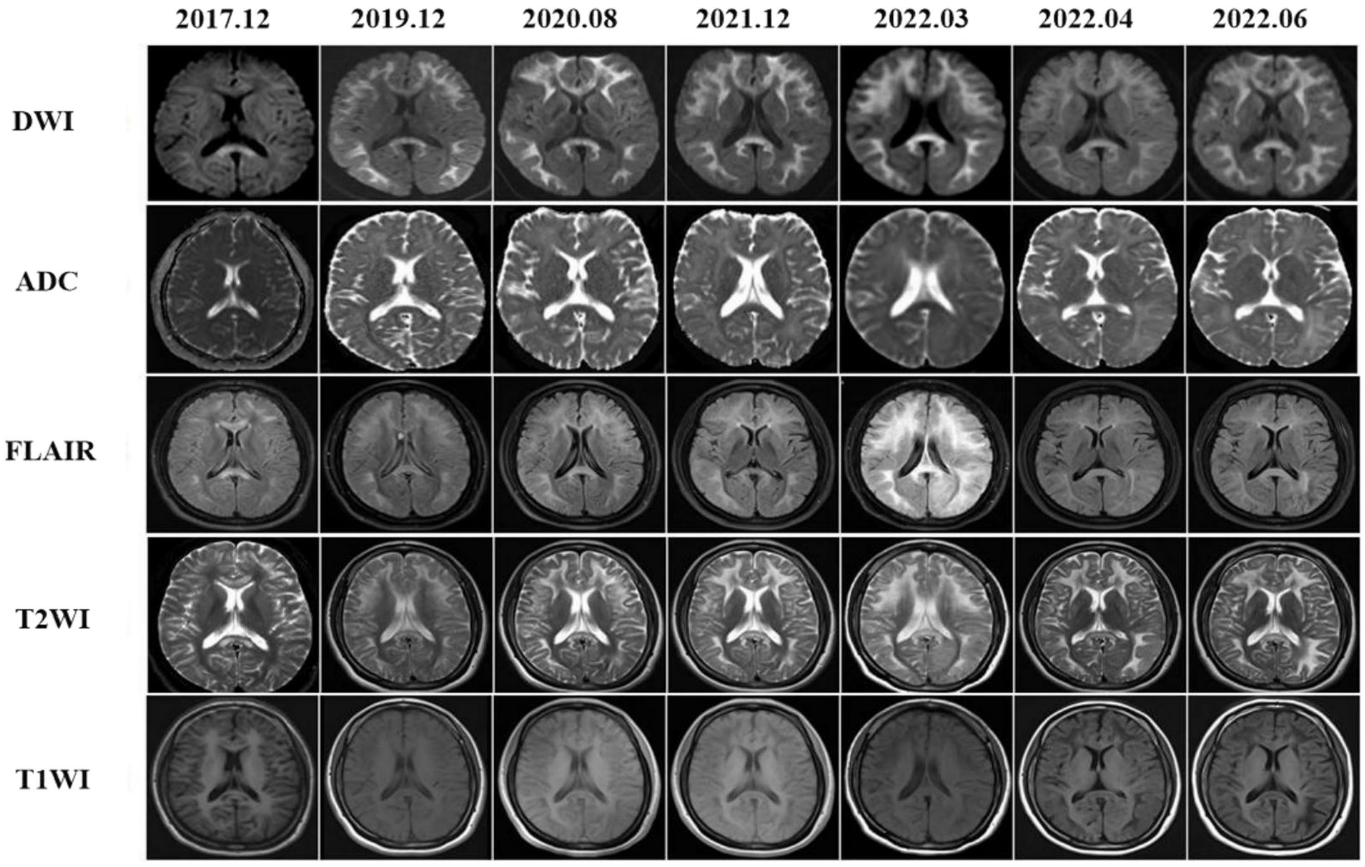
Brain MRI findings showed diffused leucoencephalopathy in T2WI and FLAIR images, there were also hyperintensities in the corpus callosum. On DWIs high intensities were noted within the CMJ, the corpus callosum and external capsules. These abnormal MRI findings were gradually expanded from the CMJ to the deep white matter that were more prominent in DWIs.

Subcortical high-signal linear lesions in the bilateral hemispheres and corpus callosum appeared on DWI at the time of the first attack, corresponding with high intensities on FLAIR (Figure 2, 2017–12). From 2019 to 2022, a series of MRI scans showed that as the disease progressed, abnormalities in the cerebral area developed along the CMJ and gradually expanded from the subcortical to periventricular regions. External capsules were also involved (Figure 2, 2022–6).

#### Patient 3

**Figure 3:**
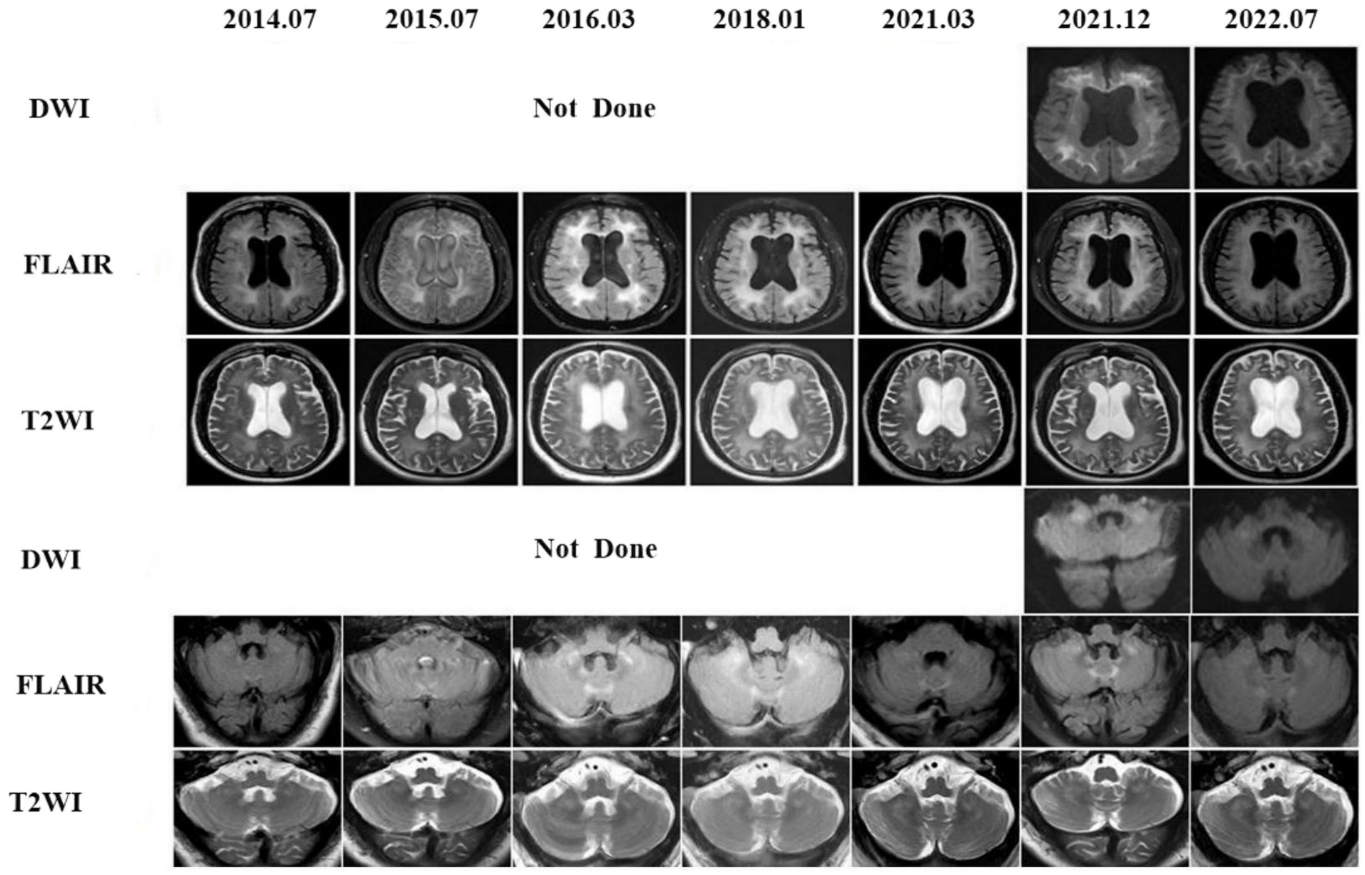
Eight years follow-up of the patient 3. The first brain DWI in our hospital was performed in 2021, high signal intensities in the CMJ, external capsules, the cerebellar white matter and paravermal regions on DWI already existed and kept stable after 1 year later. In fact, a series of conventional brain MRI images of this patient were undertaken from 2014 to 2021, showing leucoencephalopathy in those regions on FLAIR. Unfortunately DWIs were not done and the patient was misdiagnosed all the time until 2021.

On retrospectively searching his imaging data, we serendipitously found that he underwent brain MRIs at our hospital at ages 64, 65, 66, 68, and 70 years. Seven years before the onset of cerebral hemorrhage, MRI scans revealed widespread and confluent T2WI, FLAIR hyperintensities in the deep white matter (DWM), and patchy aberrant high-signal lesions within the external capsules, cerebellar hemispheres, and paravermal areas. Unfortunately, DWI was not performed during this period. As a result, the patient was misdiagnosed with a demyelinating disease until 2021. The head MRI showed confluent and bilaterally symmetrical leukoencephalopathy on T2WI and FLAIR images (Figure 3, 2021–12). High-intensity regions were also found in the CMJ and the regions around the root of the gyrus. On DWI, classic flame-like high signals were noted in the CMJ of bilateral cerebral hemispheres; patchy high signal shadows were also observed in the external capsules, the paravermal areas and cerebellar hemispheres. The lesions were relatively unchanged on DWI after 6 months.

#### Patient 4

**Figure 4:**
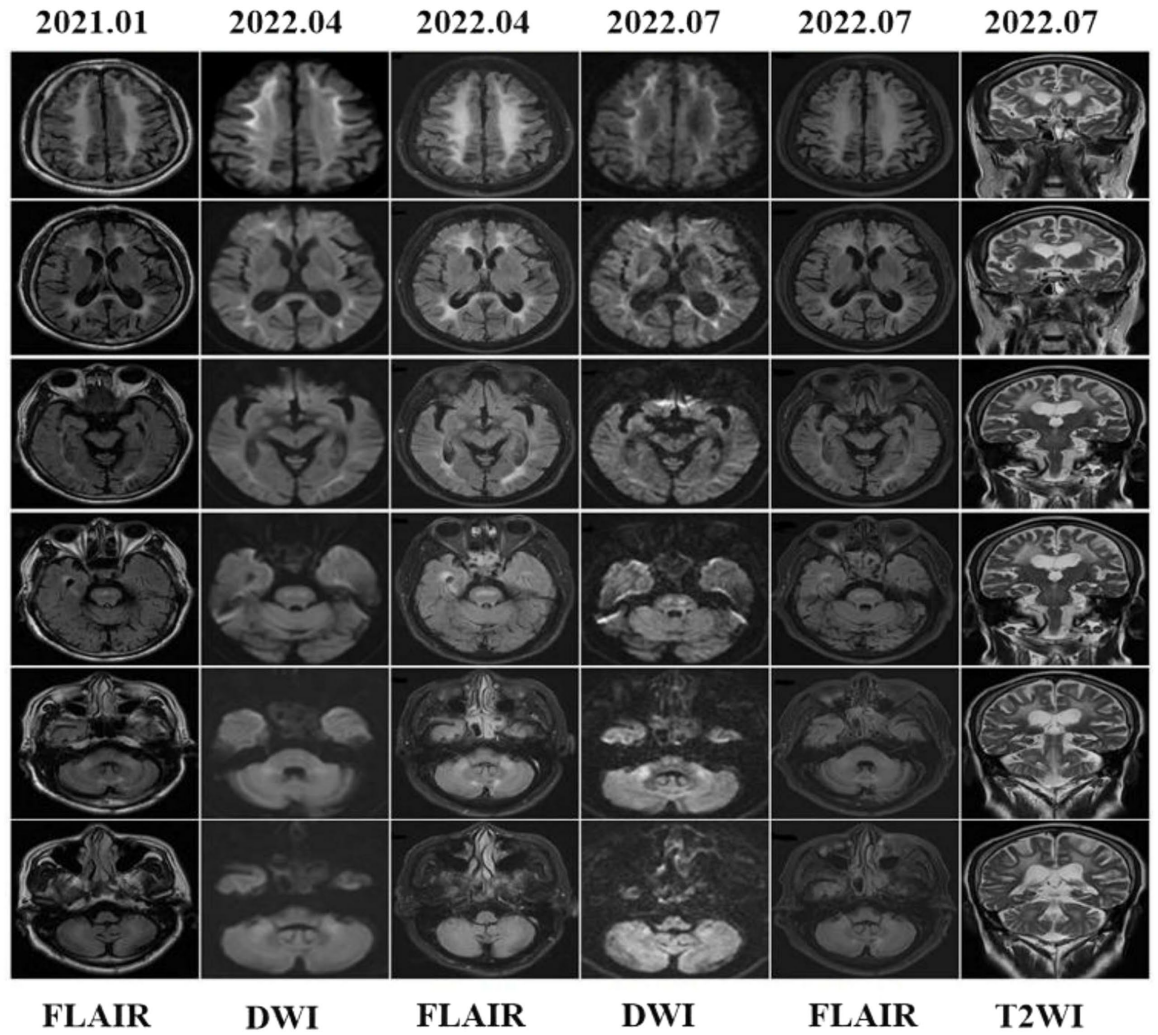
Brain MRI findings showed leucoencephalopathy in fluid attenuation recovery (FLAIR) images, but DWI was not performed in 2021. In 2022, cranial MRI showed high-intensity lesions in the CMJ, the corpus callosum, internal capsules, bilateral cerebral peduncles, middle cerebellar peduncles (MCP sign), paravermal areas and even the cerebellar white matter and brain stem. Brain stem abnormal MRI findings increased over time.

In 2021, a head MRI performed in another hospital showed diffuse and confluent high-signal shadows in both cerebral hemispheres. Furthermore, there were bilateral, extensive and confluent T2WI and FLAIR hyper-intensities in the corpus callosum, internal capsules, bilateral cerebral peduncles, brain stem, bilateral middle cerebellar peduncles (“MCP sign”), cerebellar hemispheres, and cerebellar paravermis, which all extended from the subcortical to periventricular regions. However, DWI was lacking. One and a half years later, the patient was referred to our hospital’s mental health care center for a cranial MRI. DWI showed flame-like high signals along the CMJ, and the lesions penetrated into the DWM with urine-stain-like alterations (Figure 4). In addition, the corpus callosum, internal capsules, bilateral cerebral peduncles, brain stem, bilateral middle cerebellar peduncles, cerebellar hemispheres, and cerebellar paravermal regions also displayed patchy high signals. Three months later, a repeated scan in 2022 revealed that the lesions had barely changed.

### Brain MRI longitudinal findings of the lesion severity

**Figure 5:**
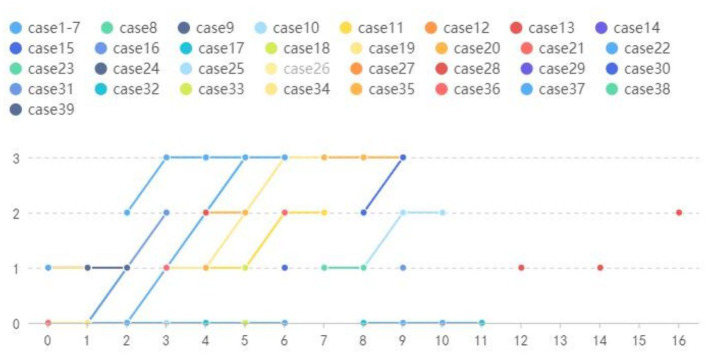
Year = follow-up time; Grade = relative changes between two MRIs; Style 1: Cases 1–7 High signal intensities in the CMJ were negative on DWI even follow-up to 11 years. Style 2: Cases 8–16 High signal intensities in the CMJ were negative on DWI at first and then typical DWI findings gradually emerged overtime. Style 3:Cases 17–19 High signal intensities in the CMJ disappeared during follow-up. Style 4: Cases 20–39 High signal intensities in the CMJ were positive at first onset and progressed gradually during the follow-up.

We included a total of 135 NIID patients, including our four patients, of whom 39 (29%) had follow-up outcomes and 96 (71%) had none. A total of 171 DWI-positive examinations and 53 DWI-negative examinations were performed (Table 2). Our results revealed four main DWI dynamic change patterns [N(number of MRI scans) ≥ 2]: seven cases in style 1 (mean follow-up time: 4.43 years): high signal intensities in the CMJ were negative on DWI even after an 11-year follow-up; nine cases in style 2 (mean follow-up time 6.22 years): high signal intensities in the CMJ were initially negative, but over time, typical DWI findings gradually emerged; three cases in style 3 (mean follow-up time 7.67 years): high signal intensities in the CMJ disappeared during follow-up; 20 cases in style 4 (mean follow-up time 5.05 years): high signal intensities in the CMJ were initially positive and continuously improved over time (Figure 5). Besides, single DWI was negative in 11 cases (*N* = 1) and positive in 85 cases (*N* = 1).

**Table 2:** Summary of DWI changes style of the NIID patients.

DWI results	DW −−	DWI −+	DWI −+−	DWI ++	DWI −	DWI +	
Numbers	*N* ≥ 2	*N* ≥ 2	*N* ≥ 2	*N* ≥ 2	*N* = 1	*N* = 1	
Cases	7	9	3	20	11	85	135
Mean follow-up time	4.43Y	6.22Y	7.67Y	5.05Y	0	0	3.90
Follow-up Times DWI+	0	19	9	58	0	85	171
Follow-up Times DWI−	21	15	6	0	11	0	53

N, number of MRI scans.

### Brain MRI longitudinal findings of the lesion location

**Figure 6:**
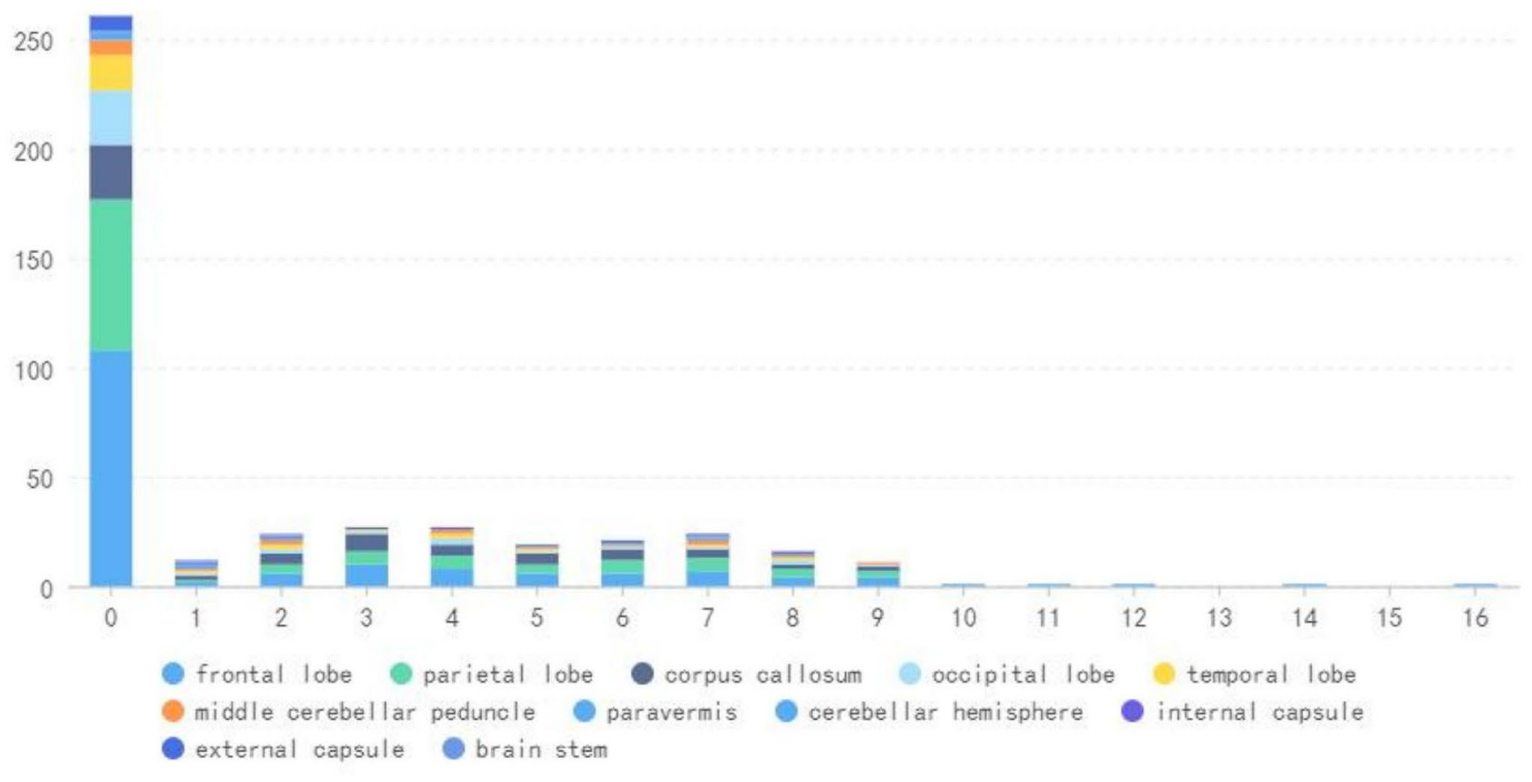
The DWI longitudinal course of the lesion location.

During the follow-up period, there were 166 (97%) positive DWI examinations in the frontal lobe, 109 (63.8%) in the parietal lobe, 63 (36.8%) in the corpus callosum, 38 (22.2%) in the occipital lobe, and 25 (14.6%) in the temporal lobe, as well as 18 (10.5%) in the middle cerebellar peduncle, 11 (6.4%) in the external capsule, 7 (4.1%) in the cerebellar hemisphere, 4 (2.3%) in the internal capsule and 4 (2.3%) in the paracerebellar vermis, and 2 (1.1%) in the brain stem (Table 3 and Figure 6).

**Table 3:** Summary of DWI longitudinal changes of location.

Year	F	P	CC	O	T	MCP	EC	CH	IC	PV	BS
0	108	69	25	25	16	7	7	0	0	3	0
1	2	1	2	1	1	1	0	1	1	1	1
2	6	4	5	2	2	2	0	1	1	1	1
3	10	6	8	1	1	0	1	0	0	0	0
4	8	6	5	3	2	2	1	0	0	0	0
5	6	4	5	1	1	1	1	0	0	0	0
6	6	6	5	1	0	1	1	0	0	0	0
7	7	6	4	1	1	2	0	1	1	1	0
8	4	4	2	2	1	1	0	1	1	1	0
9	4	3	2	1	0	1	0	0	0	0	0
10	1	0	0	0	0	0	0	0	0	0	0
11	1	0	0	0	0	0	0	0	0	0	0
12	1	0	0	0	0	0	0	0	0	0	0
13	0	0	0	0	0	0	0	0	0	0	0
14	1	0	0	0	0	0	0	0	0	0	0
15	0	0	0	0	0	0	0	0	0	0	0
16	1	0	0	0	0	0	0	0	0	0	0
Total	166	109	63	38	25	18	11	7	4	4	2

## Discussion

A notable aspect of our study is that we analyzed 224 MRI scan findings from 135 NIID patients reported in the literature, and explained the chronological changes on DWI. Four main discoveries were made in this study. First, we discovered that there were four primary DWI dynamic change patterns. Second, we found that high DWI signal lesions penetrated into the DWM in the later phrases in patients 3 and 4. Third, in patients 3 and 4, the brain stem, the bilateral cerebral peduncles, and bilateral cerebellar white matter could be affected. Finally, we confirmed that the anterior-to-posterior progression of high-signal lesions on DWI was a continuous pattern.

In our investigation, we discovered four distinct patterns of longitudinal DWI changes. In the first pattern, DWIs were negative throughout the disease duration; seven patients in the literature have this pattern. For instance, Liang et al. (11) and Wiltshire et al. (12) both described an NIID patient with no DWI abnormalities throughout a 10-year follow-up period. Furthermore, we found 11 NIID patients with negative DWI findings in a single MRI scan (13, 14). One explanation for why DWI is still negative despite the presence of multiple intranuclear inclusions and neuronal loss in the brain tissue is that the spongy degeneration has not yet occurred in the early stages. According to McFadden et al. (15), the majority of the neurons in all neocortical areas including the hippocampus had round, eosinophilic intranuclear inclusions, whereas neurons in the substantia nigra displayed substantial neuronal loss without spongy alterations.

In the second pattern, DWIs were initially negative before turning positive. Nine cases of this pattern were observed in the literature (16–18). According to Tamura et al. (19), one NIID patient’s DWIs were negative for the first 4 years before showing high signals in the CMJ of the bilateral frontal–parietal lobes. We suppose that among NIID individuals, this may be the traditional longitudinal DWI pattern. DWI characteristics were compatible with neuropathological changes (20). Additionally, in our patient 1, MRI scans initially displayed focal cortical edema and showed high signals on T2WI, FLAIR, and DWI. However, a year later, the lesions entirely disappeared without encephalatrophy and leukoencephalopathy. Interestingly, 2 years later, on DWI the CMJ in the bilateral frontal lobes, right parietal lobe, and corpus callosum showed characteristic lesions. There have not been any reports on this feature, though. The potential mechanism is vasogenic edema caused by disruption of the blood brain barrier (BBB). This theory is supported by the fact that some NIID patients with encephalitic episodes demonstrated focal brain edema on MRI with or without gadolinium enhancement (21, 22).

In the third pattern, high DWI signals disappeared during the follow-up. In the literature, three cases had shown this pattern (23–25). According to previous studies (26), a flame-like or urine-stained high signal in the CMJ on DWI was produced as the disease advanced as a result of irreversible spongiform degeneration. However, the cause of DWI high signals has not yet been identified. Possibilities include spongiform degeneration, T2 shine-through, myelin edema, and cytotoxic edema.

Ataka et al. (27) described an NIID patient with DWI high signals extending from the left to the contralateral cerebral hemisphere after 1 month. Chun et al. (28) also described one NIID patient whose lesions progressed during a period of 17 days. Therefore, we suggest that early-stage DWI high signals in some NIID patients may be caused by reversible factors such as vasogenic edema and myelin edema, rather than spongiform degeneration.

In the fourth pattern, DWIs were positive in 20 patients on the first attack. These findings are consistent with previous studies (29–31). Yokoi et al. (20) discovered that DWI high signals in the CMJ and FLAIR high signals in the DWM corresponded with pathologic spongiotic alterations and diffuse myelin pallor, respectively. Spongiotic alterations, on the other hand, had fewer inclusions. Numerous intranuclear inclusions were seen throughout the cerebral white matter and cortices.

Previous research has found that DWI high signals seldom reached into the DWM (32). Nevertheless, we observed that late-stage lesions might spread into the DWM areas. Contrary to widespread assumption, a flame-like high signal in the DWM was apparent on DWI in patient 2, and the high signal gradually extended into the DWM. DWM with urine-stain-like high signals was also found in patients 3 and 4.

Our patients 3 and 4 displayed very strong signals in the DWM of the bilateral cerebellar hemispheres, bilateral cerebral peduncles, and brainstem, which had not been described in the literature. We hypothesized that the lesions would developed along the white matter pathways. Despite the fact that myelin loss in the cerebellar white matter has been reported previously (33). We were unable to identify the pathological cause of these abnormal signals in the cerebellum, and more studies are needed to support our findings.

We also included DWI lobar distribution data and found that the frontal and parietal lobes were initially impacted by strong DWI signals (108/171, 69/171, respectively), followed by the occipital lobe (25/171), and the tempera lobe (16/171). The corpus callosum was also involved often (25/171). The middle cerebellar peduncles, external capsules, and paravermal regions were affected in the early stages, but the internal capsules, cerebral peduncles, brain stem and cerebellar white matter were seldom harmed. As the disease progressed, the frontoparietal lobes (169/171,109/171) were the most frequently affected. After more than 10-year of follow-up, only examples of frontal lobe involvement have been reported. Meanwhile, we noted isolated corpus callosum high signals (63/171) and the absence of corticomedullary lesions in the early stages. A case of NIID was documented by Jung et al. (17) and Chen et al. (25), respectively, in which the corpus callosum was the only region showing DWI high signals at the outset of the disease, and typical DWI high signals did not appear in the frontal lobe until several years of follow-up.

The study had few limitations. The evaluation of lesion progression in this study was based on subjective judgment, which was not insufficiently reliable. In the future, quantitative analysis could be performed using advanced brain function analysis tools.

## Conclusion

We discussed the longitudinal features of 135 patients with NIID on DWI. We suggest that although abnormal DWI high signals are indicative of NIID, negative findings do not exclude the diagnosis of NIID. We speculate that as the disease advances, imaging examinations will reveal dynamic changes and NIID lesions eventually involve the DWM along the white matter tract.

## Data availability statement

The original contributions presented in the study are included in the article/supplementary material, further inquiries can be directed to the corresponding author.

## Ethics statement

The studies involving human participants were reviewed and approved by the Ethics Committee of Sichuan Provincial People’s Hospital. The patients/participants provided their written informed consent to participate in this study. Written informed consent was obtained from the individual(s) for the publication of any potentially identifiable images or data included in this article.

## Author contributions

DL: conceptualization, methodology, formal analysis, and writing-original draft preparation. KC: data curation. ST: oversight and leadership responsibility for the research activity planning and execution, including mentorship external to the core team. L-LY: acquisition of the financial support for the project leading to this publication. ML: preparation, presentation of the published work, specifically visualization. Y-SW: MRI scanning. All authors contributed to the article and approved the submitted version.

## Conflict of interest

The authors declare that the research was conducted in the absence of any commercial or financial relationships that could be construed as a potential conflict of interest.

## Publisher’s note

All claims expressed in this article are solely those of the authors and do not necessarily represent those of their affiliated organizations, or those of the publisher, the editors and the reviewers. Any product that may be evaluated in this article, or claim that may be made by its manufacturer, is not guaranteed or endorsed by the publisher.

## Abbreviations

NIID: neuronal intranuclear inclusion disease
DWM: deep white matter
CMJ: corticomedullary junction
5′UTR: 5′-untranslated region
